# A Salutogenic Perspective on Critical Care Nurse's Experiences of Supervising Nurses Without Training in Intensive Care: To Comprehend, Manage, and Feel Meaning During a Crisis

**DOI:** 10.1155/jonm/2393128

**Published:** 2024-11-16

**Authors:** A. Fredholm, A. Nordin, M. Andersson, Å. Engström

**Affiliations:** ^1^Department of Health Science, Faculty of Health, Science and Technology, Karlstad University, Karlstad, Sweden; ^2^Department of Learning, Informatics, Management and Ethics, Karolinska Institutet, Solna, Sweden; ^3^Swedish Red Cross University, Huddinge, Sweden; ^4^Department of Health, Education and Technology, Division of Nursing and Medical Technology, Lulea University of Technology, Lulea, Sweden

**Keywords:** COVID-19, critical care nurses, health, sense of coherence, supervision

## Abstract

**Introduction:** Using the COVID-19 pandemic as an example of a national and international crisis, it has been possible to show how critical care nurses (CCNs) were affected by their work situation with impact on health and wellbeing. This study sought out to investigate how CCNs stress was affected and to provide some answers as to how to react and organize care in a future crisis. The specific focus was CCNs' stressors related supervision of nurses untrained in intensive care and how these were handled in a salutogenic perspective.

**Aim:** The aim of this study was to analyze CCNs' experiences of supervision of nurses without training in intensive care during the COVID-19 pandemic, and to analyze these experiences with the help of the salutogenic concept sense of coherence.

**Materials and Methods:** The phenomena under study were explored during the years of 2021–2022 through in-depth interviews and interpreted using deductive content analysis.

**Results:** By analyzing CCNs experiences of supervising nurses without training in intensive care with the lens of sense of coherence, it was possible to show in what way these concepts influenced how to cope with the demanding situation. Sense of coherence was influenced by the inevitable prioritization of patient care and nursing interventions. This prioritization caused moral distress, but was also enhanced or decreased by CCNs sense of coherence.

**Conclusion:** When recruiting and introducing new personnel in a future crisis to any field of healthcare, but particularly to the intensive care, we would, on the basis of these findings, suggest that well-established plans are vital for how to move personnel throughout the organization, and for how to introduce the field of intensive care. Plans for how to model care with the help of RNs without specialist training should be put in place. A communication plan for the organization is also of importance to enhance transparency.

## 1. Introduction

The COVID-19 pandemic was setting the stage for investigating reactions to a modern crisis. Using the COVID-19 pandemic as an example of a national and international crisis, it has been possible to show how critical care nurses (CCNs) were affected by their work situation with impact on health and wellbeing [1–3]. The COVID-19 pandemic provides illustration to how society and organizations within society—in this case the intensive care—reacted confronted with a major crisis in modern time. This article deals with the pandemic as a phenomenon per se but seeks put to investigate how intensive care was affected and to provide some answers as to how to react and organize in a future crisis with special focus on CCNs' stressors related supervision of nurses untrained in intensive care and how these were handled in a salutogenic perspective. Arabi et al. [4] point put how the COVID-19 pandemic was a reminder that besides the core duty to care, the healthcare organization also has a duty to improve and learn. Thus, the pandemic provides a unique opportunity to transform the way we can handle future crisis and other health disasters.

The COVID-19 pandemic provided a challenge for the intensive care. Previous to the pandemic, Sweden exhibited one of the Western world's lowest numbers of intensive care unit (ICU) beds with a capacity around five ICU beds per 1,00,000 inhabitants. In March 2020, the first patient with a COVID-19 diagnosis was admitted to a Swedish ICU [5]. However, to meet demands caused by the COVID-19 pandemic, pandemic measure plans were launched and Sweden was forced to double its ICU capacity [6]. This was done by incorporating nearby premises such as postoperative-, emergency-, and operating wards and by means of rapid reconstruction turning them into temporary ICUs [7]. Available resources were transferred, additional equipment was purchased when available and elective surgery and nonemergency was down scaled. ICU staffing coordination also had to be organized in a new way, for example the nurse-to-patient ratio increased from 1:1-2 to 1:3 and were sometimes ever higher [8]. To achieve this, healthcare workers were redeployed and after training also sent to ICUs for help. A total of 4222 COVID-19-diagnosed patients were cared for in Swedish ICUs in 2020 (Swedish Intensive Care Registry). The large volumes of patients to the hospitals led ICUs to implement drastic measures, changing everyday routines within a week [7]. In total, these changes had considerable impact on management of ICU patients and the everyday practice of intensive care staff in general and CCNs specifically.

The COVID-19 pandemic provided institutional constraints when the intensive care had to prioritize care and CCNs suffered moral distress. Engström and colleagues [9] showed how the COVID-19 pandemic added additional stressors to the already complex and demanding work of providing nursing care in ICUs. Furthermore, the study showed institutional constraints as an obstacle to nursing interventions, illustrating how CCNs felt forced to prioritize and not provide the nursing interventions they saw as necessary. These situations and experiences represented high levels of moral distress, and the risk for compassion fatigue was obvious. In the war against COVID-19, CCNs have been in the frontlines, lacking colleagues and equipment. Andersson et al. [10] reported high moral distress scores for instance when CCNs were part of futile care and poor teamwork. Patient-related factors induced a higher degree of moral distress than system-related factors, possibly indicating the need for CCNs to be able to provide person centered nursing care [11].

According to Antonovsky [12] the salutogenic concept of *sense of coherence* is constituted by three components: *comprehensibility, manageability*, and *meaningfulness* which can be applied to our research. The most important part of in the sense of coherence is to feel *meaningfulness*. To see that there is hope, even without guarantees for success, is according to Antonovsky [12] a part of experiencing meaningfulness. *Comprehensibility* means the ability to understand the stimuli one is exposed to and a sense that one can understand events that happen in life. Despite a sudden increase of work CCNs with high *comprehensibility* can manage an understandable situation unlike an inexplicable and chaotic situation. *Manageability* means the degree to which people feel that there are resources to cope with demands and challenges. *Meaningfulness* means a sense of being motivated and to have things that are worthwhile to invest energy in. The choice to work in ICU during the pandemic can be seen as *meaningfulness* and a step to increase the individual's sense of coherence, and a movement on the continuum from illness toward health [13]. A sense of being in control, can be understood as a part of Antonovsky's [13] *comprehensibility* when people reach control in inexplicable and chaotic situations.

By investigating if relationships between prerequisites, care environment, and person-centered processes influence CCNs' health and well-being, it might be possible to identify aspects in the work environment that require targeted interventions to reach healthy workplaces [10]. It seemed that more experienced CCNs had greater ability, despite various obstacles within and outside the ICU organization, to maintain a person-centered approach in their practice. It was also possible to show the importance and impact on health of teamwork and the possibility to create relationships with colleagues, patients and relatives. The care environment needs to facilitate person centered practice, then the potential of teamwork gets prerequisites to be fully realized. Andersson et al. [10] also showed an existing relationship between health and moral distress, both in terms of general health and of self-rated exhaustion disorder. Patient-related factors induced a higher degree of moral distress than system-related factors, possibly indicating the need for CCNs to be able to provide person centered nursing care and to create relationships as a part of a meaningful context.

Salluh et al. [14] showed how the resilience of healthcare systems have been tested, but how the COVID-19 pandemic showed how the main prerequisites for ICU resilience usually were absent. A resilient health system is defined by the capacity of stakeholders and institutions to prepare, adapt, and respond to a crisis. Furthermore, a resilient system should aim to learn from the crisis. Crisis response should be combined with better personnel management and staff wellness [14].

We know that CCNs experienced high levels of moral distress during the pandemic. By analyzing their experiences with the lens of sense of coherence we might see in what way comprehensibility, manageability, and meaningfulness are factors influencing how to cope with such demanding situations as nursing critically ill persons with COVID-19 during the pandemic when no one knew for how long this would continue. This can create important knowledge for how to structure and organize care from a micro, meso, and macro perspective in future crises.

### 1.1. Aim

The aim of this study was to analyze CCN experiences of supervision of nurses without training in intensive care during the COVID-19 pandemic, and to analyze these experiences with the help of the salutogenic concept sense of coherence.

## 2. Materials and Methods

The conducted research is methodologically positioned within the interpretative qualitative research paradigm. The phenomenon under study was explored through in-depth interviews in order to capture CCN: first-hand experiences. As such, data was interpreted openly and inductively to explore these experiences. To further interpret, deepen, and illuminate findings, a theoretical, deductive content analysis was performed according to Elo and Kyngäs [15] using the salutogenic concept of sense of coherence Antonovsky [12].

### 2.1. Setting and Participants

Swedish ICUs with a total of more than 40 patients treated in each ICU during 2020 with COVID-19 diagnoses were identified by the Swedish Intensive Care Registry. Participants were CCNs working in ICUs during the second year of the COVID-19 pandemic and met the following inclusion criteria: employed as a registered nurse and having a specialist training in intensive care and master degree in nursing science. The identified ICUs represented university hospitals, county hospitals, and local hospitals. However, during the COVID-19 pandemic, the level of care for patients suffering from COVID-19 was to a large extent the same regarding complexity and specialization.

### 2.2. Data Collection

Fifteen CCNs at nine hospitals had in one of our previous studies [11] registered their interest in participating in an interview study as well by listing their email addresses in connection to a questionnaire package. Participants were approached via email and time and date were determined for interview on a digital platform. Data were collected between March and May 2022 via digitally recorded interviews with the CCNs. This enabled compliance with Swedish social distancing requirements (Public Health Agency of Sweden, 2020) while data collection from geographically distant participants could be arranged. The ICNs chose the time for the interview. The interviews were conducted separately by three of the authors (ÅE, *n* = 1; AF, *n* = 4, and AN, *n* = 10). Data were collected by individual semistructured interviews lasting from 45 to 80 min, and these were voice-recorded and transcribed verbatim. Field notes were used and considered during the interpretation of each interview as a whole. The interview guide was constructed with open-ended questions to provide an opportunity for the ICNs to describe their own experiences. The questions revolved around their experience in intensive care units during the COVID-19 pandemic with a particular focus on the domains of organization and leadership in a crisis; inconsistent demands, ethically challenging situations and priorities during the pandemic and nursing competence, supervision of students and introduction of coworkers without intensive care training during the pandemic. Questions also revolved around their experience regarding absence of relatives to patients and personnel protective equipment during the pandemic. Follow-up questions were asked to deepen the dialogue and enable reflection. A pilot interview was conducted and after analysis decided to incorporate in findings as the interview as no alteration of worth were made in the interview guide and the interview held thick descriptions of the studied phenomena. The study was conducted in accordance with the Code of Ethics of the Declaration of Helsinki and adhered to the principles of confidentiality, integrity, right to self-determination, and privacy, as well as transparency and secure data processing. The study was given ethical approval by the Swedish Ethical Review Authority (Dnr 2020-04428). Participants received an information letter where the aim and context of the study were described, along with principles of confidentiality and their right to abort the study at any given moment.

### 2.3. Data Analysis

The phenomena under study were explored and interpreted using deductive content analysis according to Elo and Kyngäs [15] in order to discern data with explicit relation to the concepts of *comprehensibility, manageability*, and *meaningfulness* as defined and operationalized by the Swedish Institute for Stressmedicine [16].

Here, *comprehensibility* was understood as the understanding of the world as structured and arranged, and your ability to reflect on these events. In the context of a work environment, *comprehensibility* was about feeling safe and secure and to have control of the situation and its different demands. *Manageability* was understood as the perception that you have sufficient resources to cope with different events constructively. In a work environment, *manageability* was seen as the combination of resources and abilities in the individual and in the environment. Professional skills and previous experiences are important, but also the possibility to apply these skills. Access to tools to perform the work at hand is important, and such tools can be both concrete artifacts, as well as invisible tools such as methods and approaches. *Manageability* is also about physical and psychological stamina. *Meaningfulness* was seen as the sense of engagement and motivation for different events, and the ability to view these events in a larger context, but also how personally important events were. In the work environment there is need for clear goals with our actions and a clear perception of the use of these actions to experience *meaningfulness*. Fellowship and solidarity are important, and are created in meaningful relationships characterized by openness, cooperation, and trust. Initially text that related to the experience of supervising was extracted from each interview. This text was later condensed into meaning units and the meaning units were sorted into the themes relating to the concepts of comprehensibility, manageability, and meaningfulness.

## 3. Results

Four informants were men and 11 women (Table 1). All were CCNs with specialist training in intensive care and master degree in nursing science. Ages varied between 31 and 60 years of age evenly spread. Work experience in intensive care varied between 2 and 30 years with the majority having an experience of approximately 15 years. The intensive care setting was mostly in larger or middle-sized hospitals in both rural and urban settings with a varying amount of ICU beds. All ICUs where informants were active had the same level of care regarding complexity and specialization. Informants were married or living with a significant other.

**Figure 1:**
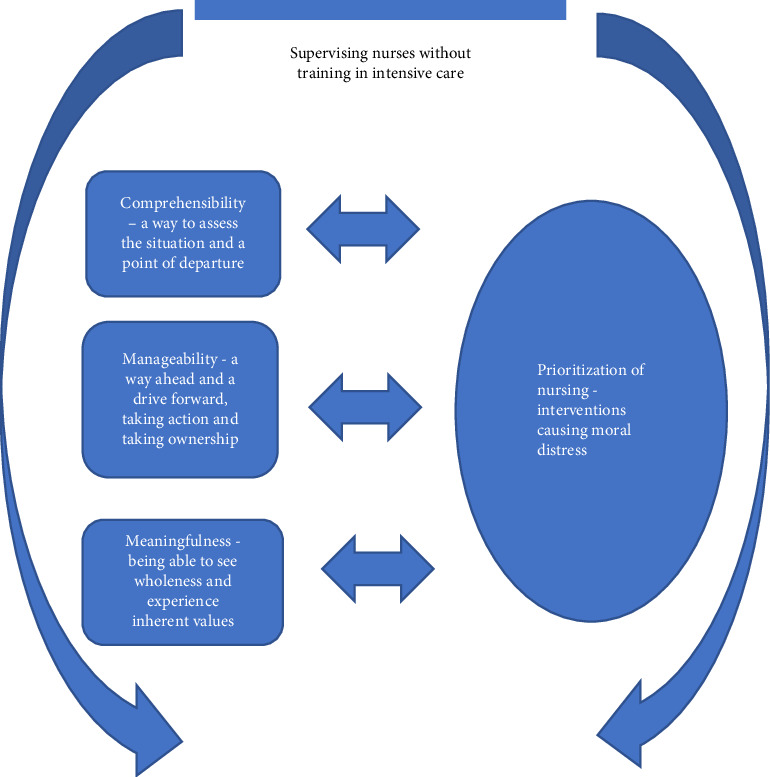
Prioritizations of care and moral distress interdependent relationship with comprehensibility, manageability, and meaningfulness seen in findings.

When deductively analyzing data with the help of the salutogenic perspective and the concepts of *comprehensibility, manageability,* and *meaningfulness* a pattern connected to prioritization of nursing interventions emerged. All prioritization of nursing interventions were retrieved from situations where CCNs supervised nurses without training in intensive care, showing how this particular circumstance had an impact on the experience of moral distress (Figure 1).

Comprehensibility, manageability, and meaningfulness were all connected to and influenced by, the inevitable prioritization of patient care and nursing interventions (Figure 1). This prioritization caused moral distress per se, but was also enhanced or decreased by CCNs ability to comprehend, manage, and perceive meaning with the situation, that is, a when CCNs understood, found ways to handle a situation, and perceived a meaning or higher purpose, the moral distress became easier to bare. As such, findings portraits a circular movement, where moral distress had an impact on comprehensibility, manageability, and meaningfulness, but where these concepts and the sense of coherence also constitutes a protecting factor against moral distress.

### 3.1. Comprehensibility—A Way to Assess the Situation and a Point of Departure

This theme was about how CCNs understood and interpreted the situation at hand, and was here seen as a point of departure, where CCNs assessed the situation and tried to understand the impact on patient care on nursing interventions, and on themselves. Here, CCNs perceived different levels of control and tried to discern one's competence and that of others.

“*It is a special satiation to nurse critically ill patients, but I have done so for many, many years, and I am very glad and grateful that I have this experience actually. And that I can discern certain things…but off course, you are being sent in to something potentially deadly, but I decided you know, but I stopped listening to the news because I couldn't take it.*” (Margareta)

CCNs tried to interpret the level of responsibility for oneself and the responsibility for others in the organization. The importance of work experience was stressed. Here, having a level of control was described as important and this was achieved by having a level of understanding of the current situation. At the same time, it was clear that the change to engage more people and hands was evident for the CCNs.

*“It'ss more like you feel like you have been doing two jobs at the same time, I mean, to introduce new staff usually, you want to do it well, and that it should be sustainable. Yes, it's a big effort to introduce someone new, but here, you knew that it could be for just a couple of weeks or maybe almost only for this shift…so much energy went in to show and explain where I really could have done it faster doing it myself, but you also knew that it meant something in the long run, that there were more of us.*” (Sarah)

### 3.2. Manageability—A Way Ahead and a Drive Forward, Taking Action, and Ownership

This theme depicted how CCNs adapted to the situation at hand and how they learned new things and tried to create understanding, structure and clarity. Here, a clear movement ahead was detected as a forward-moving process. Structures was seen as both the attempts to structure “in your own head” what was going on, but also to create structure within the organization to adapt to the situation. This structure building could consist of one's own cognitive processes, but also about more concrete things such as creating memos and try to develop the processes of care and nursing interventions.

*“The other part was that I and a colleague created a educational material because we realized that we would need more staff and that not everyone were going to be CCNs, or used to the intensive care environment either. So we created this material for this person that we imagined would come to us eventually, we did this on a few days' notice, it went very quickly and we did this before the first COVID patient even came to us.”* (Andreas)

Sometimes, however, manageability was about giving up and, painfully, having to realize one´s limitations.

*“At first, I had the ambition to help them with everything, like I would tell them how I thought and what works and why, but as time went this ambition faded, I couldn't stand it, it was not possible, and I know that on some instances it was like “well…good luck…please go ahead, go…”and this feeling was horrible, to have to back, and feel that I cannot handle any more than this…I am busy…You will have to solve the situation…”* (Mattias)

Learning was stressed as an important factor contributing to changing the situation to the better. On the down-side of taking ownership of the situation, was the sometimes-occurring demands on the profession, on the CCNs themselves, to discern and interpret guidelines (or lack thereof) of how to prioritize nursing interventions. The help from managers to prioritize interventions, and lifting this responsibility from the individual and the profession was greatly appreciated.

*“Rather quickly, you realized, and it was also said by the nurse managers, that we all need to be kind to ourselves, we have to lower the demands on ourselves and on the intensive care that we are used to provide because it is not possible now, there are harsh prioritizations…and it was really so…”* (Anna)

But, other times, the plan from management and leaders was perceived as impossible to follow, particularly in regard to the personnel situation where staff without intensive care training had to work in the ICU.

### 3.3. Meaningfulness—Being Able to see Wholeness and Experience Inherent Values

In this theme the experience of having been a part of the intensive care during the COVID-19 pandemic was valued as meaningful. A sense of having contributed was prominent together with a sense of pride. Experiences of fellowship during hard times were revealed and valued.

*“That we can handle so much more then we maybe believe, and now I am talking about the collective group of RN assistants, RNs and physicians. We rise to the occasion and take a responsibility above and beyond when needed, without further thoughts about the prize. I am really proud of having been a part of this, even with the cost for me and my colleagues. And it feels amazing that we might see an end to it, and it is something that I will carry with me for the rest of my life…much knowledge, but also many feelings and thoughts that will live with me for a long time.”* (Tobias)

The situation was expressed in terms of experiences of “war-like conditions” created meaning and justifying things like the prioritization of care. These war-like conditions created a comprehensive understanding of the whole situation. The intensive care was valued different than before, suddenly a picture of what intensive care really was and meant *at the core* emerged.

*“…For me it has become clear that…especially with the assessment I make and that it requires a specialist competence to assess such critically ill patients, and this became very clear as RNs without this competence became coming in large numbers. It is not self-evident at all, and I understood that even if I am not bedside, I still make assessments in the corner of my eye and make interventions silently and beforehand, It is very evident that you need a specialist training to do these assessments on these critically ill patients. And I have had double the amount of patients as usual, and I have always had a feeling that I´m not seeing everything with all patients, even if there has been staff bedside, but without specialist competence…”* (Sofia)

The profession and adherent professional competencies were valued differently by the CCNs who expressed a new-found admiration for the profession. The flow of new personnel to the intensive care and RNs performing tasks and interventions usually reserved for CCNs evoked a sense of fear of hollowing out nursing within intensive care with a subsequent diminishing quality of care.

*“We talked and worried about how it would become with the specialist role of the intensive care nurse (CCN, authors remark). When you engage people from all over and think that it will work. I know we discussed this some, that we were worried about that people might think that anyone can work in intensive care, like people recruited from the street…”* (Elin)

## 4. Discussion

The aim of this study was to analyze CCNs' experiences of nursing during the COVID-19 pandemic, with special focus on supervision of nurses without training in intensive care. Furthermore, the aim was to analyze these experiences with the help of the salutogenic concept sense of coherence. The findings are presented within the sense of coherence-themes. Betke, Basińska, and Andruszkiewicz [17] found in their study about relationship between sense of coherence and strategies for coping with stress within a group of nurses, that the sense of coherence serves as a health potential in a stressful working environment. A high sense of coherence translates into better mental health, correct functioning in the working environment, and using adaptive strategies of coping with stress. Masanotti et al. [18], state in their systematic review that high sense of control and meaningfulness relate to a healthy work environment for nurses.

### 4.1. Comprehensibility—A Way to Assess the Situation and a Point of Departure

In the sudden and difficult change of their work situation, CCNs felt the work situation was chaotic, but at the same time they experienced that the situation was under some kind of control, which can be interpreted as parts of comprehensibility. According to Antonovsky [13], comprehensibility means the ability to understand the stimuli one is exposed to a sense that one can understand the events in life. Bruyneel et al. [19] found that during the COVID-19 pandemic, the prevalence of unfinished nursing care in ICUs was high and the most frequently unfinished items were in the planning and communication. That can be viewed as not being comprehensibility, and if it continues might lead to feelings of meaningless and exhaustion. Engström et al. [9] found that CCNs felt that they were forced to prioritize and not provide nursing interventions they saw a need for and wanted to provide but could not. 87% of these CCNs had provided orientations for new co-workers, and 52% had supervised intensive care nursing students, 86% reported that they had prioritized nursing care differently during the pandemic and 14% had not. The qualitative analysis resulted in one theme, Institutional constraints as an obstacle for nursing interventions, with three categories: prioritizing lifesaving interventions, performing nursing interventions less frequently, and not able to provide the nursing care I wish to provide.

### 4.2. Manageability—A Way Ahead and a Drive Forward, Taking Action, and Ownership

By thinking about how to drive the work forward and take lead of action and owning the situation the CCNs developed the manageability. CCNs learned a lot and they developed nursing interventions to handle the situation, which made them cope with these unfortunate and unexpected circumstances. Manageability means the degree to which people feel that they have adequate internal and external resources to cope with demands and stressors [13]. Elements of the work environment such as professional development opportunities and a supportive organization climate appear to be crucial for encouraging nurses to stay at their workplace [20]. Al Zamel et al. [21] state that job satisfaction, work environment, and leadership style are examples of factors that are positively associated with nurses' intention stay, and suggest more qualitative studies to understand reasons that influence intention to leave or remain in the organization. The degree of manageability might influence intention to stay in the organization, and the prerequisites that are needed to increase this should be offered. Vogt et al. [22] suggest resilience-boosting interventions for CCNs in order to decreasing the likelihood of developing burnout and also decrease staff turnover.

### 4.3. Meaningfulness—Being Able to See Wholeness and Experience Inherent Values

By being a part of the intensive care nursing the CCNs felt they had contributed to something high valued, and they felt proud for this. The fact that they had worked together and experienced fellowship during these hard times was valued high, and they felt there was a meaning with their work. Meaningfulness means sense of being motivated and to have things that are worthwhile to invest energy in [13]. According to Vifladt et al. [23], a positive safety culture is associated with a strong sense of coherence and suggests that CCNs who experience stressful situations as meaningful can mediate a positive perception of the safety culture in ICUs. Nursing practice of high quality will probably be experienced as meaningful for the individual CCN.

### 4.4. Sense of Coherence

Comprehensibility, manageability, and meaningfulness together constitute a sense of coherence. In the present study, we have chosen to analyze the concepts separately; however, with an understanding that there are connections between the concepts and that they are difficult to distinguish from one another. Even though there are reasons to believe that the presence of one component such as comprehensibility, would indicate the presence of the other components, and subsequently a sense of coherence, these findings cannot sustain this notion. Betke, Basińska, and Andruszkiewicz [17] showed how the sense of coherence could serve as a health potential in a stressful working environment. Nurses with a stronger sense of coherence more often chose coping strategies and it was recognized that the sense significantly could modify an individual's functioning in a stressful working environment. Furthermore, a comprehensive literature review by Masanotti et al. [18] showed how sense of coherence provided a solid foundation for examining how work is organized. It was here proposed that nursing management should focus on creating work environments which strengthens the sense of coherence, rather than concentrating on resolving the effects of stress at an individual level. Comprehensibility was improved by a clear understanding of one's role and responsibilities and a transparent communication.

## 5. Methodological Considerations

This data in this study consists of thick descriptions with a vast amount of information on many aspects of the intensive care during the COVID-19 pandemic. However, the sections relating to the supervision of nurses not trained in intensive care stands out as especially thick and rich on data, which speaks to the trustworthiness of the findings. Interviews were conducted digitally, possibly interfering with the possibility to create a deeper relationship, and to be able to discuss sensitive issues. Our experiences though, were that the informants were all very motivated to speak about their experiences and needed no or little prompting to do so, even when it came to stories about very difficult circumstances. Also, the fact that we were three interviewers and two interpreters of data and subsequent further control of interpretations of findings in the whole research group is a testimony to the trustworthiness of the study.

The deductive approach in the analysis can be viewed as both an enrichment and a limitation. The strength in the approach lies in the enhanced transferability, and the weakness in the possible risk of missing important information.

## 6. Conclusions

In the event of a future crisis impacting the healthcare system in general, and the intensive care in particular, there are some points to be drawn from this study regarding the wellbeing of individuals, but with implications for organizations. In this study, CCNs experienced a fundamental need to understand the surrounding situation; this implies that managers and organizations in a crisis should attempt to be as transparent as possible. Manageability was perceived when there was room for action and decisions on an individual basis. However, the organization should not leave the individual alone in vital events such as for instance the prioritization of nursing interventions. Meaningfulness represented the ability to discern values in the situation, such as making a contribution for society, but also the close connection to one's fellow colleagues. When recruiting and introducing new personnel in a future crisis to any field of healthcare, but particularly to the intensive care, we would on the basis of these findings suggest that well-established plans are vital for not only how to move personnel throughout the organization, but also for how to introduce the field of intensive care. Plans for how to model care with the help of RNs without specialist training should be put in place. A communication plan for the organization is also of importance to enhance transparency. However, these findings would suggest that there is no denying the individual responses to the crisis that the COVID-19 pandemic represented, and therefore we are humble before the response and actions of individual CCNs, regardless of the support—or lack of support—from the organization.

## Figures and Tables

**Table 1:** Description of informants, *N* = 15.

Age	31–60 years
Gender	Women = 11, men = 4
Specialist training in intensive care	*n* = 15
Work experience intensive care	2-30 years, *m* = 15 years
Married or living with significant other	*n* = 15

## Data Availability

Access to data is restricted due to promises of confidentiality to participants.
